# A case-only analysis of the interaction between *N*-acetyltransferase 2 haplotypes and tobacco smoke in breast cancer etiology

**DOI:** 10.1186/bcr1013

**Published:** 2005-03-21

**Authors:** Timothy L Lash, Brian D Bradbury, Jemma B Wilk, Ann Aschengrau

**Affiliations:** 1 Boston University School of Public Health, Boston, MA, USA; 2 Boston University School of Medicine, Boston, MA, USA; 3 Amgen Inc, Thousand Oaks, CA, USA

## Abstract

**Introduction:**

*N*-acetyltransferase 2 is a polymorphic enzyme in humans. Women who possess homozygous polymorphic alleles have a slower rate of metabolic activation of aryl aromatic amines, one of the constituents of tobacco smoke that has been identified as carcinogenic. We hypothesized that women with breast cancer who were slow acetylators would be at increased risk of breast cancer associated with active and passive exposure to tobacco smoke.

**Methods:**

We used a case-only study design to evaluate departure from multiplicativity between acetylation status and smoking status. We extracted DNA from buccal cell samples collected from 502 women with incident primary breast cancer and assigned acetylation status by genotyping ten single-nucleotide polymorphisms. Information on tobacco use and breast cancer risk factors was obtained by structured interviews.

**Results:**

We observed no substantial departure from multiplicativity between acetylation status and history of ever having been an active smoking (adjusted odds ratio estimate of departure from multiplicativity = 0.9, 95% confidence interval 0.5 to 1.7) or ever having had passive residential exposure to tobacco smoke (adjusted odds ratio = 0.7, 95% confidence interval 0.4 to 1.5). The estimates for departure from multiplicativity between acetylation status and various measures of intensity, duration, and timing of active and passive tobacco exposure lacked consistency and were generally not supportive of the idea of a gene–environment interaction.

**Conclusion:**

In this, the largest case-only study to evaluate the interaction between acetylation status and active or passive exposure to tobacco smoke, we found little evidence to support the idea of a departure from multiplicativity.

## Introduction

The aromatic and heterocyclic amines are among the constituents of tobacco smoke that have been identified as carcinogens [1,2]. These carcinogens require host-mediated metabolic activation to electrophiles, which readily bind nucleophilic DNA, to induce a mutation and, ultimately, cancer [3]. Two pathways metabolize aromatic amines [4]. First, aromatic amines can be *N*-acetylated in the liver by *N*-acetyltransferase-2 (NAT2) or *N*-acetyltransferase-1 (NAT1) [5]. This is a detoxifying pathway. Second, aromatic amines and heterocyclic amines can be *N*-oxidized by P450 enzymes in the liver or in extrahepatic tissues [4]. This oxidation competes with the hepatic *N*-acetylation for aromatic amines but not for heterocyclic amines. The product of the oxidation is then either *O*-acetylated by NAT2 or NAT1, a reaction that yields the activated electrophile, or detoxified by competing enzymatic pathways [4,6]. The NAT2 enzyme therefore has a dual role: it detoxifies aromatic amines hepatically but may also play a role in activation of aromatic amines and heterocyclic amines in extrahepatic tissues such as the breast.

NAT2 is a polymorphic enzyme in humans [5,7-10]. Those who possess homozygous wild-type alleles are classified as fast acetylators – because they have a higher rate of metabolic activation of aryl aromatic amines – and those who possess certain polymorphisms are called slow acetylators because they have a lower rate of metabolic activation of these amines [9,11]. Depending on which metabolic pathway predominates at critical junctures of exposure and tissue susceptibility, fast acetylators may be at higher or lower risk of smoking-induced breast carcinogenesis than slow acetylators. Postmenopausal women who smoke and have a reduced ability to detoxify by-products of tobacco smoke, as measured by their NAT2 genotype (slow acetylators), have an excess risk of breast cancer [12-14]. In one of these studies, this excess risk was found to be limited to women who had smoked for 20 years or more [14]. In another study, the postmenopausal women who were rapid acetylators were found to be at highest risk [15]. In a fifth study, the association between smoking and breast cancer showed little dependence on acetylation rate [16].

Most epidemiologic studies that have examined the relation between active cigarette smoking and breast cancer have found weak or null associations [17,18]. A meta-analysis of the studies that excluded from the analysis those women who had been passively exposed reported that the risk of breast cancer for active smokers was more than twice as much as that for women never actively or passively exposed to tobacco smoke [19]. Studies comparing women who were passive smokers with women who had never been either active or passive smokers have also shown consistent elevations in breast cancer risk associated with smoking [20-23]. Recently, two case–control studies [24,25] have reported effect modification by acetylation status for both active and passive smokers. Both studies found stronger associations between breast cancer risk and passive exposure to smoke among rapid acetylators. Though both studies also found an association between active smoking and breast cancer risk, the magnitude of the risk was greater among slow acetylators in the study by Chang-Claude and colleagues [24] and among fast acetylators in the study by Morabia and colleagues [25]. Inconsistent findings have prevented any meaningful conclusions from being drawn about the interaction of acetylation status and exposure to tobacco smoke in the etiology of breast cancer.

We collected genetic and behavioral information from incident primary breast cancer cases arising in five different sites across the United States. We used a case-only design to examine the potential interaction between acetylation status – as assigned by NAT2 genotype – and self-reported active or passive smoking status. We hypothesized that slow acetylators would be at increased risk of breast cancer associated with both active and passive smoking, and that these risks would be more pronounced among women whose exposure began before their first pregnancy or at an early age. The case-only design is optimal for assessing multiplicative interaction when the genotype and environmental exposure are independent of one another. This investigation is the largest case-only study to examine the interaction between NAT2 acetylation status and history of tobacco exposure as it relates to the risk of breast cancer.

## Materials and methods

### Study population

The cases of female breast cancer included in this analysis were identified as parts of two study populations [26,27]. The first population included women with pathologically confirmed incident invasive breast cancer diagnosed between 1987 and 1993 among residents of eight towns on Cape Cod, Massachusetts, and that were reported to the Massachusetts Cancer Registry. The second population included women with pathologically confirmed, incident stage I, stage II, or stage IIIa breast cancer that were diagnosed from December 1996 to September 1999 at hospitals in Los Angeles, California; Rhode Island; Minnesota; and North Carolina.

### Data collection

#### Buccal cell samples for genotyping

Introductory letters were mailed to breast cancer patients in 2001 and 2002. A trained interviewer followed the letter with a telephone call to answer questions and solicit participation. Patients who agreed to participate were sent an enrollment package containing an introductory letter, summary information about the study, an informed consent form, instructions for submitting a mouthwash sample, a safety-sealed sample of mouthwash, and a wide-mouth sample-collection bottle. Participants collected the sample and returned it in a postage-paid box along with their informed consent form. Buccal cells were precipitated by centrifugation and stored at -70°C until a batch of 90 samples had been collected. Batches were sent by overnight delivery on dry ice to Qiagen Genomics (Bothell, WA, USA) for DNA extraction and genotyping.

Qiagen Genomics applied proprietary Masscode technology to measure Masscode tags, which are low-molecular-weight compounds linked to the DNA via a photocleavable linker. The tag is cleaved in flow into a mass spectrometer, and a Microsoft Access database converts the raw analytical data into statistically generated genotype calls. The assay has been validated in over one million genotypes. Existing primers were used to characterize NAT2 genotypes at ten single-nucleotide polymorphisms (SNPs) in each buccal cell sample.

The Qiagen genotyping data characterized each participant as homozygous wild-type, heterozygous, or homozygous polymorphic at each SNP. Inferred haplotypes were estimated from the genotyping data using an expectation-maximization algorithm implemented in the software program SNPHAP , and the predicted haplotypes with the highest probability were used for the primary analyses.

#### Interview data

Patients who were included in the study were interviewed on the telephone by trained interviewers using a structured interview to obtain information on demographic characteristics, history of active and passive exposure to tobacco smoke, and known or suspected risk factors for breast cancer. Patients from the Cape Cod study population were interviewed between March 1997 and March 1998. Patients from the second study population were interviewed approximately 40 months after their date of diagnosis to gather the variables primarily used in this analysis.

### Analytic variables

#### NAT2 genotype

The literature on the expression of specific SNPs in the NAT2 gene guided the phenotypic assignments for each haplotype used in this study [11,28-34]. We considered a woman a 'rapid acetylator' if she was homozygous for the NAT2*4a or NAT2*12 haplotype, an 'intermediate acetylator' if she was heterozygous for the NAT2*4a or NAT2*12 haplotype, and a 'slow acetylator' if she had any other combination of the NAT2 polymorphisms listed in Table 1.

#### Tobacco exposure

We considered a woman an active smoker if she reported smoking 100 or more cigarettes in her lifetime, and a passive smoker if she was not herself a smoker but reported living with someone who was a smoker. Women who were neither active nor passive smokers were considered separately. For women who reported having smoked 100 or more cigarettes in their lifetime or who lived with someone who smoked, information on the duration, intensity, and timing of exposure to tobacco smoke (active or passive) was also collected.

#### Covariates

In addition to information about smoking, we collected information on health and behavioral risk factors, including alcohol use, body mass index (BMI), family history of breast cancer (yes or no), history of benign breast disease, and parity. BMI was calculated as weight divided by the square of height (kg/m^2^). A woman was considered to have a first-degree family history of breast cancer if she reported that her mother, sister(s), or daughter(s) had been diagnosed with breast cancer. We defined alcohol use according to the number of drinks a woman reported 'usually' having: nondrinker, ≤ one drink/month, few drinks/month, few drinks/week, almost every day, and unknown.

### Analytic strategy

Ambrosone and colleagues [12] found that the rapid and intermediate arylamine *N*-acetyltranferase activity groups do not differ in their phenotypic expression (acetylation status). Based on this finding and others [35,36], we collapsed rapid and intermediate acetylators into the group of rapid acetylators. We examined the interaction of acetylation status and exposure to tobacco smoke among the breast cancer cases available for analysis. We used logistic regression analysis in SAS [37] to quantify departure from multiplicativity. We generated odds ratios (ORs) and 95% confidence intervals (CIs) to estimate the departure from multiplicativity between smoking status and acetylation status (gene–environment interaction). We examined the ORs separately for active and passive smokers. Women who were fast acetylators and who had never been either active or passive smokers were the reference group for all analyses. We controlled for the influence of potential breast cancer risk factors including age at diagnosis of breast cancer, alcohol consumption, BMI, first-degree family history of breast cancer, geographic location (state where breast cancer diagnosis was made), and history of benign breast disease using multiple variable logistic regression.

We also evaluated departure from multiplicativity for variables describing the duration, intensity, and timing of active smoking or exposure to passive smoking. For active smokers, we examined the ORs in categories of the number of packs of cigarettes smoked per day, duration of smoking, age at onset of smoking, when a woman began smoking in relation to the first live birth of a child, and time since cessation of smoking. For passive smokers, we examined the duration of passive exposure, age when first passive exposure began, and when this first passive exposure occurred in relation to the first live birth of a child.

## Results

Among the Cape Cod population, 330 of 483 eligible women agreed to receive a sample collection kit; the remainder refused or could not be contacted. Of the 330 who received a kit, 272 returned a sample and 269 samples yielded DNA that could be genotyped. Among the second study population (from California, Rhode Island, Minnesota, and North Carolina), 372 of 410 eligible women agreed to receive a sample collection kit and the remainder refused or were unable to be contacted. Of the 372 who received a kit, 321 returned a sample and 233 had samples that yielded DNA that could be genotyped and had the requisite interview data. In both studies, 56% of eligible participants were genotyped and included in the analysis. The proportion of smokers among nonparticipants was not significantly different from that among participants in either study population. The mean age was greater among nonparticipants than among participants (mean ages 66 years versus 61, respectively, in the Cape Cod population, *P *= 0.0001; and 74 versus 73 in the second study population, *P *= 0.03), reflecting greater losses to follow-up among older women. Age was not associated with genotype among the participants. The proportion of participants who were slow acetylators, active smokers, and passive smokers did not vary significantly with their site of enrollment. Among the genotyped controls in the Cape Cod study, the OR for association of acetylation status (fast versus slow) with exposure to tobacco smoke (women who had ever smoked actively versus all the others studied) was 1.06 (*P *= 0.90). This finding is consistent with those of earlier studies [12,14-17,24,25], in which acetylation status and active smoking were not significantly associated among controls.

Table 2 provides demographic and risk factor characteristics for the 502 breast cancer patients in the analytic sample according to acetylation status. The distribution of age, family history of breast cancer, and history of benign breast disease was nearly identical for fast and slow acetylators. There were small differences in alcohol consumption and BMI between fast and slow acetylators and a noticeable difference in the proportion of women who had had one to three live births (46% versus 61%, respectively). The great majority of the participants (97%) were white (data not shown).

We observed no substantial departure from multiplicativity between acetylation status and history of ever having smoked actively (adjusted OR estimate of departure from multiplicativity (AOR) = 0.9) or of ever having experienced passive residential exposure to tobacco smoke (AOR = 0.7) (Table 3, which also shows confidence intervals). The ratios of the upper limits of the intervals to their lower limits were about 3 and 3.7 for the crude and adjusted estimates of effect, respectively (Table 3). These ratios measure the precision of the estimates of effect and indicate adequate precision about these estimates.

Estimates for the departure from multiplicativity between acetylation status and the various measures of intensity, duration, and timing of active and passive tobacco exposure are presented in Table 4. For active smokers, we found estimates lacking consistent directionality. The AOR estimates for women in the categories with the highest intensity (packs/day) and greatest length (in years) of smoking were in opposite directions. For example, the departure from multiplicativity was above the null for women who had smoked two or more packs per day (AOR = 1.8) but below the null for women who had smoked for 40 or more years (AOR = 0.7). For the variables describing the age at which a woman began smoking, when she began smoking in relation to her first live birth, and the time elapsed since she quit smoking, we observed estimates of departure from multiplicativity both above and below the null.

The estimates for departure from multiplicativity between acetylation status and the measures of duration and timing of passive exposure to tobacco also lacked consistency. For the variable describing the duration of passive exposure to tobacco in the residence, we observed null and less than null associations with slow acetylation status: AOR = 1.0 for <20 years, 0.6 for 20 to <40 years, and 0.8 for 40+ years. We observed a departure from multiplicativity between slow acetylation status and passive exposure occurring exclusively before a woman's first live birth (AOR = 1.9), and a positive departure for women whose first passive exposure to tobacco smoke occurred between the ages of 12 and 20 (AOR = 2.4).

## Discussion

In this, the largest case-only study to evaluate the interaction between acetylation status and exposure to tobacco smoke, we found little evidence to support a departure from multiplicativity between acetylation status and a history of active smoking for women with breast cancer. There is some suggestion that women who were slow acetylators were at higher risk from passive exposure to tobacco smoke before their first live birth than women who had never been either passive or active smokers. A similar positive departure was observed for women who were first passively exposed between the ages of 12 and 20. The effect estimates observed in this study for measures of intensity, duration, and timing of exposure showed no consistent pattern and in some instances were statistically unstable.

Our study is one of only a few to assess the interaction between exposure to tobacco smoke and acetylation status in relation to breast cancer risk. Hunter and colleagues [16], in addition to examining the association between slow acetylation status and the risk of breast cancer, for which they reported a null association, found no evidence of an interaction between recent smoking status and NAT2 acetylation status among 706 postmenopausal women (cases and controls). Recently, two studies that removed passive smokers from the analysis of the unexposed group found suggestions of an interaction between tobacco exposure and acetylation status. Both reported a greater breast cancer risk among passive smokers who were fast acetylators [24,25]. The findings among active smokers were not consistent, however. Morabia and colleagues [25] found that active smokers who were fast acetylators were at greater risk, whereas Chang-Claude and colleagues [24] found the greater risk from active smoking among slow acetylators. By parsing their cases into contingency tables (genotype by smoking group) and applying a case-only analysis, we obtained estimates of departure from multiplicativity for both studies very similar to ours, but with wider CIs.

In the only other case-only analysis, Ambrosone and colleagues [12] found a strong positive departure from multiplicativity between acetylation status and smoking at an early age (<18) and for smoking 20 or more cigarettes 20 years previously. Overall, we found departures from multiplicativity between acetylation status in relation to both active and passive exposure to tobacco smoke below the null. In subanalyses, we did find a positive departure from multiplicativity between acetylation status and smoking initiation between 14 and 15 years of age, and, separately, for first passive exposure to tobacco smoke between the ages of 12 and 20. Both findings are consistent with the hypothesis that environmental insults to developing breast tissue may increase the tissue's susceptibility to carcinogenesis, and thus may increase a woman's risk of breast cancer [38,39]. However, the lack of a consistent directionality to our estimates for the other age-at-initiation categories (≤ 13 and 16 to 17 years) suggests that these may be chance findings.

To date, numerous polymorphisms on the NAT2 gene have been identified (Table 1), which has furthered our understanding of NAT2 phenotypes and improved our ability to assign acetylation status to the breast cancer cases in this study. The genotyping procedures employed in this analysis are more accurate than the PCR-RFLP (PCR–restriction fragment length polymorphism) techniques used in previous studies [12,16,25]. Consequently, the rates of misclassification of acetylation status in this study should be less than in those studies.

Misclassification of either the genetic or environmental variables involved in an assessment of interaction by case–control design can give rise to the appearance of interaction when, in fact, there is none [40]. Our analysis of interaction using case-only data provides greater control over the impact of potential misclassification errors, because there are only two variables that are susceptible to misclassification – acetylation status and smoking status. If the misclassification rates are nondifferential, as one would expect, then the estimates of departure from multiplicativity will be biased towards the null [41]. As discussed above, in the previous case–control analyses, the impact of misclassification is less predictable. It is therefore possible that findings from previous studies evaluating the interaction of acetylation status and exposure to tobacco smoke in relation to breast cancer risk may have generated spurious estimates of interaction, even if the misclassification was nondifferential. As discussed above, case-only estimates derived from these studies were similar to ours. The attenuation of the interaction after reanalysis using the case-only design further suggests that the published case–control results may have been more susceptible to misclassification. By genotyping more SNPs with a more accurate method and by implementing a case-only design, our analysis provides a more valid assessment of the multiplicative interaction between NAT2 genotype and exposure to tobacco smoke in relation to breast cancer.

Weighing against this advantage of the case-only design is the limitation that only departure from multiplicativity can be assessed. Many epidemiologists weigh departure from additive interaction more heavily, arguing that the additive scale corresponds better to the biologic meaning of synergistic effects [42]. A further limitation of the case-only design is its reliance on the assumption that the genetic polymorphisms and environmental exposure are independent of one another [43]. Violations of this assumption can substantially distort the estimates of interaction. However, NAT2 polymorphisms and smoking history were not associated among the genotyped controls in the Cape Cod study or among the controls in earlier studies [12,14-17,24,25]. The absence of association supports the assumption of independence required to validly estimate departure from multiplicativity with the case-only design.

These results must be interpreted with the following additional limitations in mind. First, only 56% of eligible cases were available for analysis. Participation was not related to smoking status and although participation was related to age, age was not related to genotype. We expect that the selection of participants introduced no substantial bias, although we acknowledge that our study of breast cancer survivors may have influenced the estimates of effect in ways that we are unable to anticipate. Second, haplotypes were inferred from genotyping data by assigning the haplotype with the maximum probability to each case. Forty-one percent of haplotype assignments had probabilities of 100% and 91% had probabilities of 80% or better. Less than 5% had probabilities of less than 50%. We expect that the procedure used to infer haplotypes introduced little error.

## Conclusion

This large case-only analysis is the first to be able to assign acetylation status on the basis of ten SNPs. No previous analysis assigned acetylation status on the basis of more than four. In addition, the study involved the largest number of breast cancer cases used to investigate the interaction between NAT2 acetylation status and exposure to tobacco smoke as related to breast cancer risk. The combination of the most complete genotyping data and the large case-only design provides important advantages, the results of which do not suggest any substantial interaction between acetylation status and exposure to tobacco smoke in the etiology of breast cancer. Weighing against the null result is the potential for an unanticipated bias towards the null to have arisen by selection of breast cancer survivors from among the incident cases.

## Abbreviations

AOR = adjusted odds ratio estimate of departure from multiplicativity; BMI = body mass index; CI = confidence interval; NAT = *N*-acetyltransferase; OR = odds ratio; PCR–RFLP = polymerase chain reaction–restriction fragment length polymorphism; SNPs = single-nucleotide polymorphisms.

## Competing interests

The author(s) declare that they have no competing interests.

## Authors' contributions

TL conceived of the study, collected genotyping samples, participated in data analysis, and drafted the manuscript. BB conducted data analysis and drafted the manuscript. JW inferred haplotypes from the genotyping data using SNPHAP and drafted the manuscript. AA collected interview data from the Cape Cod population and drafted the manuscript. All authors read and approved the final manuscript.

## Figures and Tables

**Table 1:** Distribution of NAT2 haplotypes and their gene product acetylator phenotype in 502 breast cancer patients

Acetylator phenotype			Haplotype	References
Status	No.	%		
Fast	24	4.8	NAT2*4/NAT2*4	31–34
Intermediate	158	31.5	All intermediate	14
	148	93.7	NAT2*4/-	35
	10	6.3	NAT2*12a/-	
Slow	320	63.7	All slow	14,33,36
	81	24.6	NAT2*5b/NAT2*5b	
	139	43.4	NAT2*5b/NAT2*6a	
	37	11.2	NAT2*5b/-	14,31–33,36
	43	13.1	NAT2*6a/NAT2*6a	
	18	6.1	NAT2*6a/-	14,36,37
	2	3.3	NAT2*5c/-	

-, any other haplotype yielding heterozygosity; NAT, *N*-acetyltransferase.

**Table 2:** Distribution of breast cancer risk factors according to arylamine *N*-acetyltransferase activity (acetylation status)

	NAT2			
			
	Fast	Slow		
			
Characteristic	No. (%)	No. (%)	OR	95% CI
Age (years)				
<50^a^	22 (12)	38 (12)	1.0	-
50 to 59	15 (8)	28 (9)	1.1	0.5 to 2.4
60 to 69	59 (32)	103 (32)	1.0	0.5 to 1.9
70+	86 (47)	151 (47)	1.0	0.6 to 1.8
Alcohol use				
Nondrinker^a^	45 (14)	32 (18)	1.0	-
≤ 1 drink/month	75 (23)	43 (24)	1.2	0.7 to 2.2
Few drinks/month	77 (24)	39 (21)	1.4	0.8 to 2.5
Few drinks/week	76 (24)	39 (21)	1.4	0.8 to 2.5
Almost every day	40 (12)	24 (13)	1.2	0.6 to 2.3
Unknown	7 (2)	5 (3)	1.0	0.3 to 3.4
Body mass index (BMI) (kg/m^2^)				
<20.0^a^	30 (17)	59 (19)	1.0	-
20.0 to 24.9	114 (65)	196 (62)	0.9	0.5 to 1.4
25.0 to 29.9	24 (14)	50 (16)	1.1	0.5 to 2.0
30.0+	8 (4)	11 (3)	0.7	0.3 to 1.9
First-degree family history of breast cancer^b^				
No^a^	140 (78)	240 (77)	1.0	-
Yes	39 (22)	72 (23)	1.1	0.7 to 1.7
Parity				
Nulliparous^a^	72 (23)	27 (15)	1.0	-
1 live birth	22 (7)	22 (12)	0.4	0.2 to 0.8
2 live births	56 (17)	43 (24)	0.5	0.3 to 0.9
3 live births	70 (22)	48 (25)	0.5	0.3 to 1.0
4 live births	46 (14)	21 (12)	0.8	0.4 to 1.6
5 or more live births	54 (17)	21 (12)	1.0	0.5 to 1.9
Geographical location				
Cape Cod, Massachusetts^a^	91 (50)	178 (56)	1.0	-
Los Angeles, California	27 (15)	33 (10)	0.6	0.3 to 1.1
Rhode Island	17 (9)	38 (12)	1.1	0.6 to 2.1
Minnesota	28 (15)	34 (11)	0.6	0.3 to 1.1
North Carolina	19 (10)	37 (12)	1.0	0.5 to 1.8
History of benign breast disease				
No^a^	116 (65)	206 (65)	1.0	-
Yes	63 (35)	110 (35)	1.0	0.7 to 1.4

^a^Reference level. ^b^Includes a woman's mother, sister(s), and/or daughter(s). -, not calculated; CI, confidence interval; NAT, *N*-acetyltransferase; OR, odds ratio.

**Table 3:** Departure from multiplicativity between acetylation status and smoking status among patients with breast cancer

	Acetylation status		Crude statistic		Adjusted statistic^a^	
	
Smoking status	Slow	Fast	OR	95% CI	OR	95% CI
Active smoker	162	85	1.04	0.59 to 1.82	0.88	0.45 to 1.70
Passive smoker	114	73	0.85	0.48 to 1.52	0.75	0.39 to 1.45
Nonsmoker	44	24	1.0	-	1.0	-

^a^Controlling for age, alcohol consumption, body mass index, family history of breast cancer, family history of benign breast disease, parity, and geographical location. -, not calculated; CI, confidence interval; OR, odds ratio.

**Table 4:** Departure from multiplicativity between acetylation status and smoking status among patients with breast cancer

	Acetylation status				
				
Smoking	Slow	Fast	OR	Adjusted OR^a^	Adjusted 95%CI
***Nonsmokers***	44	24	1.0	1.0	-
***Active smokers***					
Packs (per day)					
<1	96	47	1.11	0.97	0.48 to 1.95
1 to <2	53	33	0.88	0.74	0.35 to 1.60
≥ 2	12	2	3.27	1.80	0.33 to 9.81
Data missing	1	3			
Duration (years)					
<20	39	29	0.73	0.59	0.26 to 1.35
20 to <40	79	25	1.72	1.32	0.62 to 2.81
40+	43	30	0.78	0.74	0.33 to 1.63
Data missing	1	1			
Duration (years) in relation to first birth					
Nulliparous	41	11	2.03	1.05	0.32 to 3.41
All before first	11	8	0.75	0.60	0.17 to 2.10
Before and after first	90	48	1.02	1.03	0.48 to 2.20
All after first	19	17	0.61	0.52	0.19 to 1.37
Data missing	1	1			
Age started (years)					
≤ 13	4	3	0.73	0.63	0.11 to 3.48
14 to 15	22	7	1.71	1.94	0.61 to 6.19
16 to 17	35	18	1.06	0.79	0.32 to 1.94
18 to 21	67	35	1.04	0.94	0.45 to 1.99
22 to 29	19	11	0.94	0.84	0.31 to 2.30
≥ 30	14	10	0.76	0.60	0.21 to 1.71
Data missing	1	1			
Quit before diagnosis date (years)					
Current or <5	32	12	1.45	1.25	0.50 to 3.19
5 to 15	37	19	1.06	0.82	0.35 to 1.95
>15	71	42	0.92	0.86	0.41 to 1.80
Data missing	22	12			
***Passive smokers***					
Duration (years)^b^					
<20	34	18	1.03	0.99	0.43 to 2.33
20 to <40	48	36	0.73	0.61	0.29 to 1.28
40+	29	18	0.88	0.81	0.33 to 2.01
Data missing	3	1			
Duration (years) in relation to first birth					
Nulliparous	20	14	0.78	0.52	0.16 to 1.69
All before first	20	8	1.36	1.85	0.59 to 5.83
Before and after first	56	34	0.90	0.91	0.39 to 2.10
All after first	6	5	0.65	0.39	0.09 to 1.73
Data missing	12	12			
First lived with smoker (age in years)					
<12	51	35	0.84	0.83	0.29 to 2.34
12 to 20	18	5	3.11	2.43	0.41 to 14.3
>20	35	28	0.89	0.48	0.17 to 1.42
Data missing	10	5			

^a^Controlling for age at diagnosis, alcohol use, BMI, first-degree family history of breast cancer, history of benign breast disease, and parity. ^b^Length of time lived with a smoker. -, not calculated; CI, confidence interval; OR, odds ratio.
